# Genome Wide Identification of Novel Long Non-coding RNAs and Their Potential Associations With Milk Proteins in Chinese Holstein Cows

**DOI:** 10.3389/fgene.2018.00281

**Published:** 2018-07-30

**Authors:** Wentao Cai, Cong Li, Shuli Liu, Chenghao Zhou, Hongwei Yin, Jiuzhou Song, Qin Zhang, Shengli Zhang

**Affiliations:** ^1^Key Laboratory of Animal Genetics, Breeding and Reproduction, Ministry of Agriculture and National Engineering Laboratory for Animal Breeding, College of Animal Science and Technology, China Agricultural University, Beijing, China; ^2^Shaanxi Key Laboratory of Molecular Biology for Agriculture, College of Animal Science and Technology, Northwest A&F University, Yangling, China; ^3^Department of Animal and Avian Science, University of Maryland, College Park, MD, United States

**Keywords:** long non-coding RNA, mammary gland, transcriptome, milk proteins, integrate study

## Abstract

Long non-coding RNAs (lncRNAs) have emerged as a novel class of regulatory molecules involved in various biological processes. However, their role in milk performance is unknown. Here, whole transcriptome RNA sequencing was used to generate the lncRNA transcriptome profiles in mammary tissue samples from 6 Chinese Holstein cows with 3 extremely high and 3 low milk protein percentage phenotypes. In this study, 6,450 lncRNA transcripts were identified through 5 stringent steps and filtration by coding potential. In total, 31 lncRNAs and 18 novel genes were identified to be differentially expressed in high milk protein samples (HP) relative to low milk protein samples (LP), respectively. Differentially expressed lncRNAs were selected to predict target genes through bioinformatics analysis, followed by the integration of differentially expressed mRNA data, gene function, gene ontology (GO) and pathway, genome wide association study (GWAS) and quantitative trait locus (QTL) information, as well as network analysis to further characterize potential interactions. Several lncRNAs were found (such as XLOC_059976) that could be used as candidate markers for milk protein content prediction. This is the first study to perform global expression profiling of lncRNAs and mRNAs related to milk protein traits in dairy cows. These results provide important information and insights into the synthesis of milk proteins, and potential targets for the future improvement of milk quality.

## Introduction

Milk proteins are among the most important nutrients for human, therefore, these proteins could serve as valuable indices to evaluate the quality of milk. The amount and composition of proteins in milk are largely determined by the genetic factors of the cows (Auldist et al., 2004). Although some causal genes and mutations for milk yield and composition have been identified using QTL mapping, candidate gene analysis, GWAS or NGS technologies in dairy cows (Georges et al., 1995; Andersson, 2009; Canovas et al., 2010; Wickramasinghe et al., 2011), the synthesis and secretion of milk proteins involve complex processes, and need to be thoroughly examined. It has been demonstrated that the milk production traits are under strong epigenetic regulation (Singh et al., 2012). Mechanisms of epigenetic gene regulation function through the modulation of chromatin structure, and may either repress or enhance gene expression. It has been reported that milk proteins are in fact influenced by DNA methylation (Nguyen et al., 2014; Liu et al., 2017), histone modifications, such as acetylation, ubiquitination, and phosphorylation (Wang et al., 2017), and microRNAs (Li R. et al., 2016; Wang et al., 2016). However, a new epigenetic component – long non-coding RNAs (lncRNAs), which were recently demonstrated to be critical gene regulators of cellular metabolism (Zhao and Lin, 2015), are yet to be thoroughly examined.

Long non-coding RNAs (lncRNAs), which are at least 200 bases in length, and do not possess protein-coding capabilities (Rinn and Chang, 2012), have received much attention in the past decade. It has been shown that some lncRNAs regulate the expression of genes in close proximity (*cis*-acting) or at a distance (*trans*-acting) in the genome *via* different mechanisms, including the modification of promoter activities by nucleosome repositioning (Rinn et al., 2007; Zhao et al., 2008), histone modification (Pandey et al., 2008; Khalil et al., 2009), DNA methylation (Lai and Shiekhattar, 2014; Merry et al., 2015), activating/gathering/transporting of accessory proteins, epigenetic silencing, and repression (Ponting et al., 2009; Kornienko et al., 2013). Some lncRNAs are believed to be precursor molecules that are processed into small RNAs, while others have been demonstrated to function as intact, long molecules that participate in a range of regulatory roles (Cesana et al., 2011). Increasing evidence supports the notion that lncRNAs are associated with developmental, metabolic and immunological regulation, as well as adaptations and phenotypic variation of complex traits in domestic animals (Qu and Adelson, 2012; Weikard et al., 2013; Zhou et al., 2014; He et al., 2015; Ren et al., 2016; Tong et al., 2017). Recent advances in sequencing technologies have opened a new horizon for the identification and annotation of this class of RNAs in many species (Esteve-Codina et al., 2011; Li T. et al., 2012). Although lncRNAs have emerged as a novel class of regulatory molecules involved in various biological processes, their expression profile and role in the regulation of milk protein synthesis in dairy cattle remains unknown.

In the current study, we performed whole transcriptome ssRNA-seq to examine the mammary tissue transcriptome and identify lncRNAs from Chinese Holstein cows with extremely high and low milk protein levels during peak lactation. Our study seeks to systematically identify lncRNAs potentially involved in milk protein synthesis, reveal expression profiles of lncRNAs in Chinese Holstein mammary glands and provide the resources to effectively explore their functional roles in the improvement of milk quality.

## Materials and Methods

### Ethics Statement

All procedures pertaining to the handling of experimental animals were conducted in accordance with and approved by the Animal Welfare Committee of China Agricultural University (Permit Number: DK996). All efforts were made to minimize discomfort and suffering.

### Experimental Design and Sample Collection

Based on the DHI data, 6 multiparous and healthy, mastitis-free Chinese Holstein cows with 3 extremely high and 3 low phenotypic values for milk protein percentage (high ≥ 3.5% and low ≤ 3.0%) at approximately 60 days postpartum (peak lactation) were selected for the study (**Supplementary Table S1**). The cows selected in this study were the same with our previous cows in peak lactation (Li C. et al., 2016). The cows at the Beijing Sanyuan Dairy Farm Center (Beijing, China) were maintained in free stall housing, and were fed a total mixed ration (TMR) with *ad libitum* access to water. Mammary samples were collected from the 6 cows via biopsies, as previously described (Li C. et al., 2016). All tissue samples (∼500 mg) were snap-frozen in liquid nitrogen and stored at -80°C for further testing.

### Total RNA Extraction, Library Preparation, and Illumina Sequencing

Total RNA was extracted from each mammary tissue sample using TRIzol (Invitrogen, Carlsbad, CA, United States) according to the manufacturer’s protocol with an average concentration 1,018.5 ng/μl (**Supplementary Table S2**). RNA quantity and quality were assessed using the Qubit^®^ RNA Assay Kit in a Qubit^®^ 2.0 Flurometer (Life Technologies, Camarillo, CA, United States) and by 1% agarose gel electrophoresis along with the RNA Nano 6000 Assay Kit of the Bioanalyzer 2100 system (Agilent Technologies, Santa Clara, CA, United States), respectively. All 6 samples had an RIN value greater than 7.0 (**Supplementary Table S2**). Different from previous study (Li C. et al., 2016), libraries were constructed using ribosomal RNA removal methods. Ribosomal RNA of the 6 RNA samples was removed using the Epicentre Ribo-zero^®^ rRNA Removal Kit (Epicentre, United States). Strand-specific sequencing libraries were constructed following a previously described protocol (Borodina et al., 2011). Finally, the libraries were sequenced on an Illumina Hiseq 2500 platform, which generated 125 bp paired-end reads. The sequencing data have been submitted to the NCBI Sequence Read Archive (SRA), and are accessible through the accession number PRJNA416150.

### Quality Analysis, Mapping, and Transcriptome Assembly

The resulting directional 125 bp paired-end reads were assessed for quality using FastQC. Clean reads were obtained by removing contaminating adapter molecules, reads containing poly-N, and low-quality reads (parameter -q 36 -p 90) in the raw data using the Fastx_toolkit (0.0.13). All downstream analyses were based on the high quality clean data. Sequencing reads in the FASTQ format were aligned to the bovine genome (UMD 3.1), and novel splice junctions were automatically determined using TopHat2 (version 1.4.1) (Kim et al., 2013). The default parameters were used for the analysis, except for the “-G” option together with Gene Transfer Format (GTF) file of Ensembl gene annotation (UMD3.1.85) and “–library-type = fr-firststrand”. The sampled alignment data were then fed to Cufflinks, Scripture, Stringtie and Transcomb to assemble aligned reads into transcripts (Guttman et al., 2010; Trapnell et al., 2012; Pertea et al., 2015; Liu et al., 2016). Cufflinks (version 2.02) was run with “min-frags-per-transfrag = 0”, “–library-type = fr-firststrand” and “–mask-file = ncRNA.gtf”), the ncRNA.gtf contains all the known rRNA, tRNA, snRNA and snoRNA annotations in the bovine genome. Scripture (beta2) was run with default parameters (with an omission of the “-pairedEnd” option), StringTie (version 1.0.1) was run with the parameters (-f 0.01 -c 0.01). Transcomb (V1.0) was run using the default parameters, except for “-s first”, “-l 200” and “-e 50”.

### Identification of lncRNAs

All transcripts identified using Cufflinks, Scripture, Stringtie and Transcomb were independently matched and guided by the Ensembl gene models of Cuffcompare. The transcripts with the same start, end position and exon–intron boundary, which were supported by at least two assembly programs or occurred in at least two samples, were extracted as stringent transcripts. The novel transcripts were then filtered and assembled in order to obtain putative transcripts, as previously reported (Cabili et al., 2011; Pauli et al., 2012), according to the following steps:

Step (1) Transcripts that were likely to be assembly artifacts or PCR run-on fragments according to class code annotated by Cuffcompare were removed. Among the different classes, only those annotated by ‘i’, ‘u’ and ‘x’ were retained, which represent novel intergenic, intronic, and *cis*-antisense transcripts, respectively. Transcripts with class code “=” annotated by Cuffcompare were considered as known genes.
Step (2) To avoid incomplete assemble and too many splicing events, transcripts with length ≥ 200 nt, exon ≥ 2 were retained.
Step (3) FPKM ≥ 0.3 were retained. Extremely low expression was generally considered to be transcriptional noise.
Step (4) Maximum ORF lengths of less than 120 amino acids (360 nt) were obtained by TransDecoder (3.0.1)^1^.
Step (5) Transcripts with predicted protein-coding potential were removed (protein-coding potential criteria: CPC score > 0, PLEK score > 0, and CNCI score > 0).
Step (6) All transcripts were translated into amino acid sequences through all three reading frames to remove transcripts that contain known protein domains. HMMER (Finn et al., 2011) was used to identify any known protein domain by searching against the Pfam database (Pfam 30.0) (Finn et al., 2008). Transcripts with significant Pfam hits were excluded.

Finally, those without coding potential made up the candidate sets of lncRNAs. In addition, transcripts with long ORF lengths (more than 120 amino acids) or having protein-coding potential were considered as novel protein-coding genes.

### Conservative Analysis

PhyloFit was applied to compute phylogenetic models for conserved and non-conserved regions among species. The model and HMM transition parameters were entered into phastCons in order to compute a set of conservation scores of lncRNAs and coding genes (Siepel et al., 2005). PhastCons scores were downloaded from the UCSC database. To assign a conservation score to a transcript, the median phastCons score for the concatenated exonic regions of each transcript model was calculated. The conservation score was compared among the protein-coding sequences, lncRNAs, and 5000 random genomic sequences. To generate a set of random sequences, the non-gap genome region was initially taken as the full set, and coding exons from all known and computational gene models were excluded. Next, the sequences with lengths identical to those of the lncRNAs under investigation were randomly drawn. The lncRNAs were annotated to the NONCODE database using BLASTN with *p*-value < 1e-06 (McGinnis and Madden, 2004).

### Differential Expression Analysis

The expression levels of known genes, novel genes, and lncRNAs were calculated in fragments per kilo-base of exon per 10^6^ mapped fragments (FPKM) using Cuffdiff, which provides statistical routines for determining differentially expressed known genes, novel genes and lncRNAs through the use of a model based on the negative binomial distribution (Trapnell et al., 2012). Genes or lncRNAs with a Benjamini-Hochberg (BH) adjusted *p*-value < 0.05 were designated as differentially expressed. Hierarchical clustering was performed to visualize the expression patterns of differentially expressed lncRNAs among the samples.

### Gene Ontology and Pathway Analysis

Gene ontology enrichment analysis of DEGs or lncRNA target genes were performed in DAVID database (Huang et al., 2008). BH method was used to adjust significant *p*-values. GO terms with *p*-value < 0.05 were considered to be significantly enriched. The Ingenuity Pathway Analysis (IPA) with fisher exact test method was used to test the statistical pathways enrichment of lncRNA correlated genes (Krämer et al., 2013). Pathways with -log2(*p*-value) > 1.3 were considered to be significantly enriched.

### Interaction Between lncRNAs and miRNAs

To explore whether some lncRNAs were precursors of miRNAs, miRNAs published in miRBase (Release 21)^2^ were aligned to the sequences of lncRNAs in order to find the known miRNA precursors using BLASTN with 100% match. Prediction of secondary structures for the lncRNA transcripts was made using the Vienna RNA package in the RNAfold program. The prediction software miRanda was applied to predict the interaction between miRNAs and lncRNAs with score > 160, energy < -15.

### Target Gene Prediction and Functional Analysis

The *cis* role of lncRNAs was defined as those exerting effects on neighboring target genes (Guil and Esteller, 2012). Coding genes located in both 10 and 100 Kb upstream and downstream of lncRNAs were checked using in-house Perl scripts. The *trans-acting correlation* of lncRNA and mRNA was used to identify each other through the expression level (Derrien et al., 2012). The expressed correlations between lncRNAs and coding genes were calculated using the Pearson method with *p*-value < 0.05. The QTL information of milk protein traits were extracted from the AnimalQTLdb.^3^ The 972 significant SNPs associated with milk protein traits were collected from 12 previous GWAS studies (**Supplementary Table S3**). The functional analysis was conducted based on their positions on chromosome using our in-house Perl scripts. Circos and Cytoscape softwares were used to plot integrated results, and candidate lncRNA-mRNA network, respectively (Shannon et al., 2003; Krzywinski et al., 2009).

### Quantitative Real-Time PCR (qRT-PCR)

RNA samples, the same sources and concentrations with library preparation (**Supplementary Table S2**), were reverse-transcribed to cDNA using the PrimeScript RT reagent Kit with gDNA Eraser (TaKaRa) according to the manufacturer’s instructions. Primers for qRT-PCR were designed using Primer 5.0, their specificity and complementarity were assessed using NCBI BLAST algorithm. QRT-PCR was run in triplicate using the LightCycler^®^ 480 SYBR Green I Master Kit (Roche). A reaction volume of 15 μL containing 7.5 μL of SYBR Green PCR Master Mix, 0.75 μL of forward and reverse primers (10 mM), 2 μL of cDNA, and 4.75 μL of double-distilled water. The qRT-PCR reaction conditions were: denaturation at 95°C for 10 min, 45 cycles of 95°C for 10 s, 60°C for 10 s, and 72°C for 10 s. A final extension of 72°C for 6 min was included. The results were normalized to *GAPDH* and *MARVELD1* expression to obtain ΔC_t_ values (Saremi et al., 2012; Li C. et al., 2016). Fold changes in expression were calculated using the 2^-ΔΔCt^ method. Differences in gene expression levels between HP samples and LP samples were analyzed using a Student’s *t*-test, with *p*-value < 0.05 considered to be statistically significant.

## Results

### High-Throughput Sequencing

After quality trimming and adaptor removal of the Illumina reads, an average of 128 million clean reads (range: 120 – 137 million) for each sample were obtained by ssRNA-seq (**Supplementary Table S4**). An average of 91.55% (range: 91.10–92.02%) of the reads were mapped to the bovine genome (Ensembl UMD3.1) using Tophat2. Of these, 83.22% (range: 82.01–84.41%) were uniquely mapped reads and 8.33% (range: 7.17–9.44%) were multi-mapped reads (**Supplementary Table S4**). The proportion of reads aligning to mRNAs, miscRNAs, ncRNAs, precursor RNAs, pseudogenes, rRNAs and tRNAs are presented in **Supplementary Figure S1**. It was observed that most reads were matched with mRNAs (51% - 58%), whereas between 33 and 39% of the reads were mapped outside of annotated loci, which potentially harbored the promising lncRNA transcripts.

### Genome Wide Identification and Characterization of lncRNAs

**FIGURE 1:**
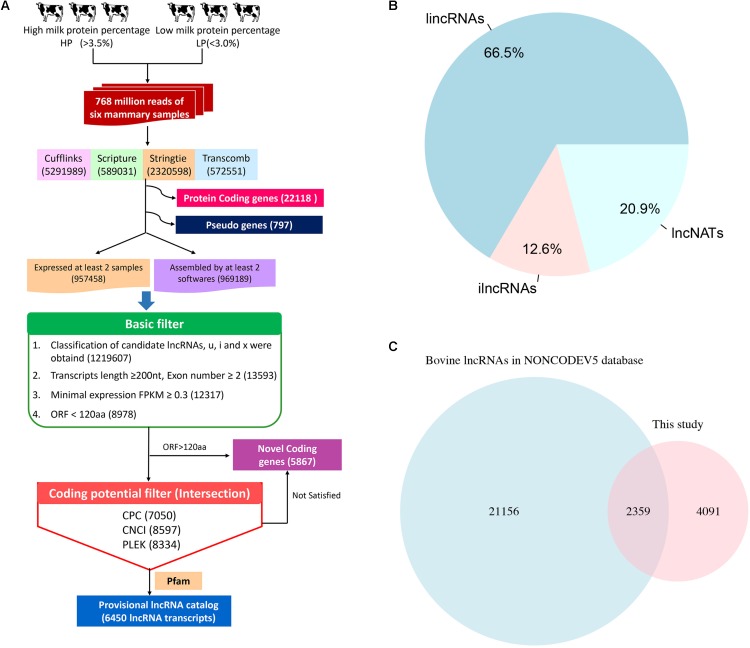
The computational pipeline used to identify lncRNA genes from RNA-seq data. **(A)** The pipeline of lncRNA identification. **(B)** The category of lncRNAs. **(C)** The result of lncRNAs overlapping with NONCODE Database.

To identify lncRNAs related to milk protein synthesis, we employed a computational approach using different filter criteria (**Figure 1A**). First, 5,291,989, 589,031, 2,320,598, and 572,551 transcripts in the 6 samples were obtained using Cufflinks, Scripture, StringTie and Transcomb, respectively, which includes all possible RNA types like protein-coding genes, novel genes, lncRNAs and pseudogenes (**Supplementary Figure S2**). After merging the 4 assemblers, a total of 1,926,647 transcripts were obtained that were detected by at least 2 assemblers in a given sample or were identified in at least 2 individual samples by the same assembler (details in Materials and Methods). Of these, 1,219,607 transcripts with class codes ‘i’, ‘u’, ‘x’, representing ilncRNAs, lincRNAs, and lncNATs were retained, respectively. Next, transcripts that were shorter than 200 bp in total length and possessed only one single exon, with a FPKM < 0.3, as well as those predicted to harbor maximum lengths of ORFs longer than 120 amino acids were filtered. Transcripts with a CPC score > 0, a CNCI score > 0, a PLEK score > 0 or overlapping with the Pfam database were then excluded. Finally, a total of 6,450 lncRNA transcripts in 5,256 lncRNA loci were identified among the 6 Chinese Holstein cows through our computational pipeline (**Supplementary Table S5**).

Among the 6,450 identified lncRNA transcripts, 4,292 and 810 were lincRNAs and ilncRNAs, respectively, while 1,348 lncRNAs flanked a protein-coding gene in a divergent orientation (lncNATs) (**Figure 1B**). The genomic coordinates of the identified lncRNA transcripts are provided in **Supplementary Table S4**. Using the BLAST algorithm, a total of 2,359 lncRNAs were found in bovine NONCODE v5.0 database (**Figure 1C**). In addition, we detected 5,867 novel mRNA transcripts in 4093 loci without any Ensemble annotation (**Supplementary Table S6**).

**FIGURE 2:**
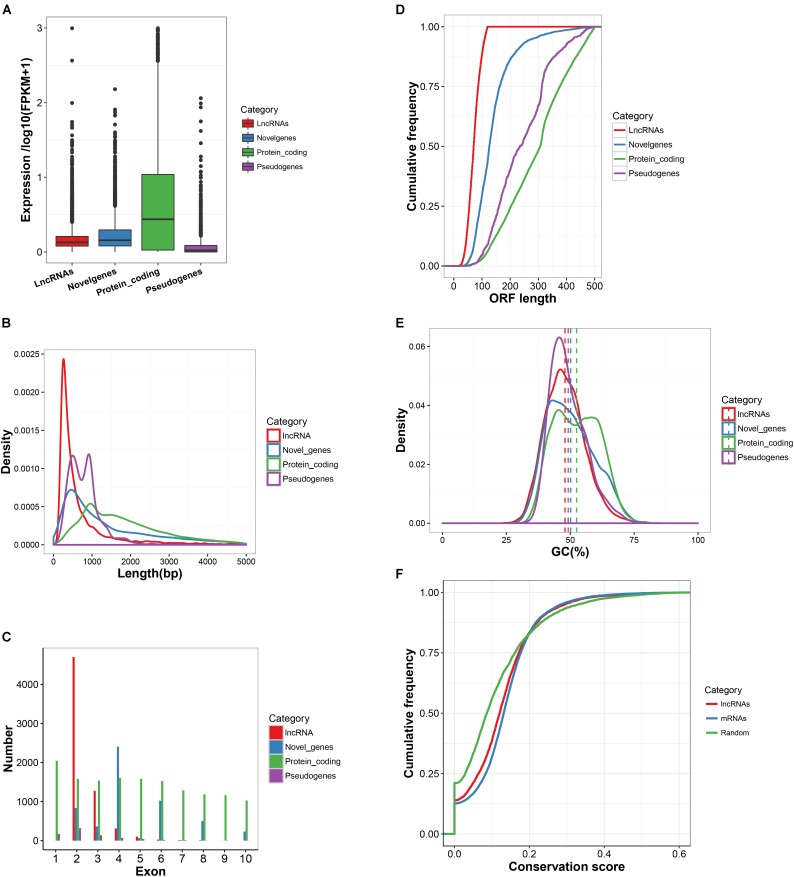
Basic features of lncRNAs. **(A)** Expression level of lncRNA transcripts, marked by red box. **(B)** Length distribution of lncRNA transcripts, marked by red line in the Figure. **(C)** The number of exons for lncRNA transcripts. As marked by a red bar line in the Figure, on the average there are 2.39 exons per transcript. **(D)** The ORF length of lncRNA transcripts compared to protein-coding genes, novel genes and pseudo genes. **(E)** The GC content of lncRNAs transcripts compared to protein-coding genes, novel genes and pseudo genes. **(F)** LncRNA sequence conservation measured by phastCons scores. lncRNAs show intermediate sequence conservation as compared to random region and protein-coding genes.

The characteristics of expression, length, exon number, structure and conservation of sequence of the newly obtained 6,450 lncRNA transcripts, 5,867 novel mRNA transcripts, 22,118 known mRNAs and 797 pseudogenes are shown in **Figure 2**. Most of the detected lncRNAs were expressed at low levels (**Figure 2A**). The size of lncRNAs was notably smaller than that of protein-coding transcripts, novel genes and pseudogenes (**Figures 2B,C**). The length of the ORFs of protein-coding RNAs (543.6 AAs) was significantly longer than those observed in lncRNAs (77.1 AAs), novel genes (153.6 AAs) and pseudogenes (266.8 AAs) (**Figure 2D**). However, approximately 4% of ORF lengths of protein-coding RNAs were observed to be shorter than 120 AAs. LncRNAs and pseudogenes exhibited lower GC contents with 47.9 and 49.2%, respectively, as compared to that of protein-coding RNAs (52.5%) and novel genes (50.2%) (**Figure 2E**). PhastCons score demonstrated that lncRNAs were less conserved than protein-coding regions but were much more conserved than random size-matched intergenic regions (**Figure 2F**). In addition, a search of the sequences from 6,450 lncRNAs against the lncRNAs from 5 other mammalian species (Human, Mouse, Rat, Pig, and Chicken) in the NONCODE v5.0 database was conducted. The conservation of lncRNA sequences across the species was limited (**Supplementary Table S7**).

### Differential Expression Analysis

**FIGURE 3:**
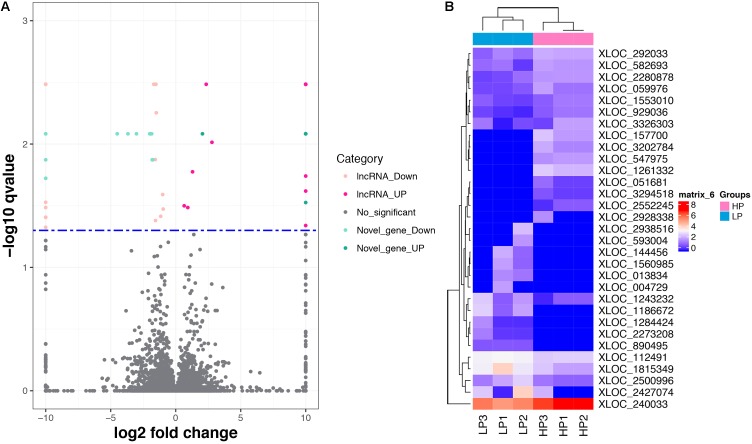
The differentially expressed lncRNAs and mRNAs. **(A)** Volcano plot displaying differentially expressed lncRNAs and mRNAs within two different comparison groups. The red and orange dots represent the differentially expressed lncRNAs (*q* < 0.05); the light blue and blue dots represent the differential expressed mRNAs (*q* < 0.05); the gray dots represent transcripts whose expression levels did not reach statistical significance (*q* > 0.05). **(B)** Cluster analysis of differentially expressed lncRNAs.

A total of 31 lncRNA genes were differentially expressed in HP relative to LP, including 15 up-regulated and 16 down-regulated lncRNAs (**Figure 3A** and **Supplementary Table S8a**, *q*-value < 0.05). Meanwhile, 18 significantly dysregulated novel genes were identified, including 6 up-regulated and 12 down-regulated novel genes (**Figure 3A** and **Supplementary Table S8b**, *q*-value < 0.05). Among the 31 differentially expressed lncRNAs, 8 lncRNAs and 10 lncRNAs were only expressed in either the HP or LP group, respectively (**Table 1**). The other 13 lncRNAs, with 5 being up-regulated and 8 down-regulated are listed in **Table 2**. Cluster analysis of differentially expressed lncRNAs are depicted in a heatmap (**Figure 3B**).

**Table 1:** The differential lncRNAs specifically expressed in HP group or LP group.

lncRNA gene	lncRNA transcript	Position	HP_FPKM	LP_FPKM	*P*-value	*Q*-value
XLOC_004729	TCONS_0023641	1:125572871-125585794	0	0.927357	5.00E-05	0.00328028
XLOC_013834	TCONS_0089292	1:56905028-56948667	0	1.19118	0.0017	0.0457879
XLOC_1186672	TCONS_1844622	6:72896473-72900508	0	3.07993	0.00045	0.0181805
XLOC_1284424	TCONS_2040856	8:65415622-65417104	0	0.903789	0.00065	0.0240837
XLOC_144456	TCONS_0207836	10:102838283-102839042	0	1.52439	5.00E-05	0.00328028
XLOC_1560985	TCONS_2392159	20:51729259-51767218	0	1.29975	5.00E-05	0.00328028
XLOC_2273208	TCONS_3400346	3:87803076-87803851	0	0.820661	5.00E-05	0.00328028
XLOC_2938516	TCONS_4397837	7:36583792-36603493	0	1.40913	5.00E-05	0.00328028
XLOC_593004	TCONS_1139318	12:75887263-75896281	0	0.829202	5.00E-05	0.00328028
XLOC_890495	TCONS_1424422	28:44935176-44936429	0	0.849668	5.00E-05	0.00328028
XLOC_051681	TCONS_0229895	1:83581354-83582143	0.870136	0	5.00E-05	0.00328028
XLOC_1261332	TCONS_2499784	19:39080345-39080701	3.5376	0	0.001	0.0327277
XLOC_157700	TCONS_0265438	11:43723923-43734354	2.41705	0	0.00135	0.0392433
XLOC_2552245	TCONS_3954401	5:31711309-31754936	1.17274	0	5.00E-05	0.00328028
XLOC_2928338	TCONS_4440543	7:21632727-21642878	2.14752	0	0.00135	0.0392433
XLOC_3202784	TCONS_6404733	9:39279279-39285358	1.74985	0	0.00135	0.0392433
XLOC_3294518	TCONS_5106036	X:4233697-4243280	0.814726	0	0.00085	0.0296505
XLOC_547975	TCONS_1098704	12:1470993-1491509	4.52107	0	0.0018	0.0474099

*HP, high milk protein percentage group in peak lactation; LP, low milk protein percentage group in peak lactation; FPKM, fragments per kilobase million.*

**Table 2:** The differential lncRNAs expressed in both HP and LP group.

lncRNA gene	lncRNA transcript	Position	HP_FPKM	LP_FPKM	Log2(Fold change)	*P*-value	*Q*-value
XLOC_582693	TCONS_0918942	2:115673519-115682184	3.31278	1.02906	−1.68671	5.00E-05	0.00328028
XLOC_059976	TCONS_0458010	5:32243816-32270339	2.05417	0.690516	−1.57281	0.0003	0.0133664
XLOC_929036	TCONS_1690898	15:65891595-65896543	1.0145	0.346711	−1.54897	0.00145	0.0416424
XLOC_2280878	TCONS_4547736	3:56909914-56937217	2.39104	0.832575	−1.52199	5.00E-05	0.00328028
XLOC_3326303	TCONS_6686797	GJ060358.1:32-6493	2.15126	0.767271	−1.48737	0.0001	0.00558672
XLOC_1553010	TCONS_3078081	20:12598342-12605288	1.48105	0.665365	−1.1544	0.0013	0.038494
XLOC_292033	TCONS_0432200	10:26697899-26702683	2.79144	1.39491	−1.00084	0.0007	0.0256703
XLOC_240033	TCONS_0542901	10:48376509-48648893	68.8414	35.3762	−0.960497	0.00105	0.0336706
XLOC_112491	TCONS_0385499	1:19929165-20182412	5.13451	8.07422	0.653097	0.00095	0.0316711
XLOC_1815349	TCONS_3601672	23:17507725-17522447	5.00749	9.53541	0.929207	0.001	0.0327277
XLOC_2500996	TCONS_4893811	4:33260787-33262913	1.35118	3.31447	1.29456	0.0004	0.0168259
XLOC_2427074	TCONS_4920751	4:36158377-36159816	1.25597	6.38418	2.3457	5.00E-05	0.00328028
XLOC_1243232	TCONS_1975869, TCONS_1991976	7:38449464-38502848	0.408342	2.81355	2.78454	0.0002	0.00966351

*HP, high milk protein percentage group in peak lactation; LP, low milk protein percentage group in peak lactation; FPKM, fragments per kilobase million.*

**FIGURE 4:**
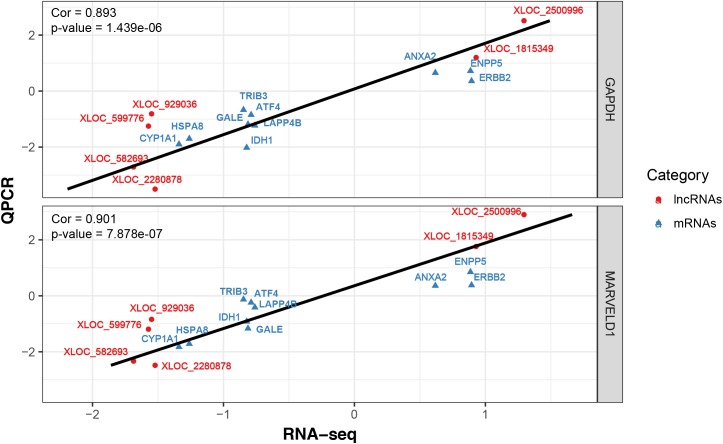
Correlations of gene expression level of 10 differentially expressed genes and 6 lncRNAs between high and low milk protein percentage using RNA-Seq and qRT-PCR. The *x*- and *y*-axis show the log_2_ (ratio of mRNA levels) measured by RNA-seq and qRT-PCR, respectively. *GAPDH* and *MARVELD1* gene were used as housekeeping internal control. The red and blue dots represent the differentially expressed genes and lncRNAs, respectively.

To validate the RNA-seq results, 10 differentially expressed mRNAs (*ANXA2, TRIB3, ATF4, IDH1, LARP4B, GALE, HSPA8, ERBB2, CYP1A1* and *ENPP5*) and 6 lncRNAs (XLOC_582693, XLOC_0599776, XLOC_929036, XLOC_2280878, XLOC_1815349 and XLOC_2500996) were randomly selected for qRT-PCR analysis. In **Figure 4**, the relative fold changes in expression detected by qRT-PCR are presented, and were found to be consistent with the RNA-seq data (*R* = 0.931 and 0.873). This indicated that our transcript identification and abundance estimation were highly reliable.

### Prediction of Relations Between lncRNAs and miRNAs

To determine whether lncRNAs are in fact precursors of miRNAs, we compared the lncRNA sequences and miRNA sequences that obtained from miRbase, and found that 13 lncRNAs harbored 8 complete miRNA precursors (**Supplementary Table S9**). Prediction of the secondary structures of lncRNA transcripts indicated that some lncRNAs contained a stable hairpin structure for miRNA precursors. For example, XLOC_1223728 harbored bta-mir-2887-2 (**Supplementary Figure S3**). To investigate whether the identified lncRNAs were targeted by miRNAs, we analyzed the 6,450 lncRNA transcripts using miRanda. A total of 4,972 lncRNA transcripts were predicted to be targeted by 788 bovine miRNAs (**Supplementary Table S10a**). Of these, 206 lncRNAs were targeted by miR-15a, miR-486, miR-135, miR-101a, miR-152 and miR-139, which were reported to involve in milk protein synthesis (**Supplementary Table S10b**). Interestingly, one differentially expressed lncRNA (XLOC_059976) was predicted to be targeted by miR-139 and miR-152, which implied XLOC_059976 could act as a regulatory factor for the process of milk protein synthesis.

### Correlation of Expression Levels Between lncRNAs and Protein-Coding Genes

To understand the possible biological roles of the lncRNAs in bovine milk protein synthesis, we investigated co-expression patterns of lncRNA genes and protein-coding genes in these 6 mammary gland samples. *Trans-acting correlations* of expression between lncRNAs and protein-coding genes were examined first. We found 5,251 lncRNAs were significantly correlated with 18,227 mRNAs (*p*-value < 0.05), including 31 differentially expressed lncRNAs that significantly correlated with 11,161 mRNAs (*p*-value < 0.05, **Supplementary Table S11**).

A search for protein-coding genes within 10 kb upstream/downstream and 100 k upstream/downstream of the lncRNAs were conducted. It was revealed that 3,369 protein-coding genes resided within a range of 10 kb of 3,065 lncRNAs, as well 9,656 protein-coding genes resided within a range of 100 kb of 4,535 lncRNAs (**Supplementary Table S12a**). Of particular interest is the observation that 7 lncRNA transcripts (TCONS_4241670, TCONS_0484807, TCONS_0484809, TCONS_1820871, TCONS_1859003, TCONS_1845219, TCONS_5606159) were detected in close proximity to casein genes such as *CSN1S1, CSN1S2, CSN2* and *CSN3*. Furthermore, TCONS_0484809 (XLOC_061135), an intronic lncRNA, is located in the first intron of the *CSN3* gene, indicating that milk protein synthesis may be regulated by the action of lncRNAs on neighboring protein-coding genes (**Supplementary Figure S4**).

Interestingly, we found the expression in both 10 and 100 kb upstream/downstream genes of lncRNAs were observed to be higher than random protein genes. Moreover, the closer the neighboring genes were to lncRNAs, the higher the co-expression would be (10k > 100k, **Supplementary Figure S5**). Within the 100 kb upstream and downstream of lncRNAs, 979 neighboring protein-coding genes were detected to be significantly correlated with 867 lncRNAs (**Supplementary Table S12b**). GO analysis demonstrated these 979 neighboring genes were involved in regulation of transcription, mammary gland branching involved in thelarche, negative regulation of transcription from RNA polymerase II promoter, positive regulation of tyrosine phosphorylation of Stat5 protein and peptide catabolic process (**Supplementary Table S13a**). IPA showed that these target genes of lncRNAs were significantly enriched in 141 pathways, many pathways were associated with the process of protein synthesis, such as EIF2 signaling, JAK/Stat signaling, GnRH signaling, PRL signaling, mTOR signaling and growth hormone signaling (**Supplementary Table S13b**). In addition, we observed seven differentially expressed protein-coding genes were in close proximity of 5 differentially expressed lncRNA genes. In summary, 6 lncRNA-mRNA gene pairs were regulated in the same direction, including 5 down-down pairs and 1 up-up pair, and 1 pair inversely. Three target genes (ENSBTAG00000032428, *CCDC184* and *ASB8*) for XLOC_059976 were regulated in the same direction, and one target gene (*PRR15L*) for XLOC_1261332 was regulated inversely (**Supplementary Table S12c**).

### Integrated Analysis

**FIGURE 5:**
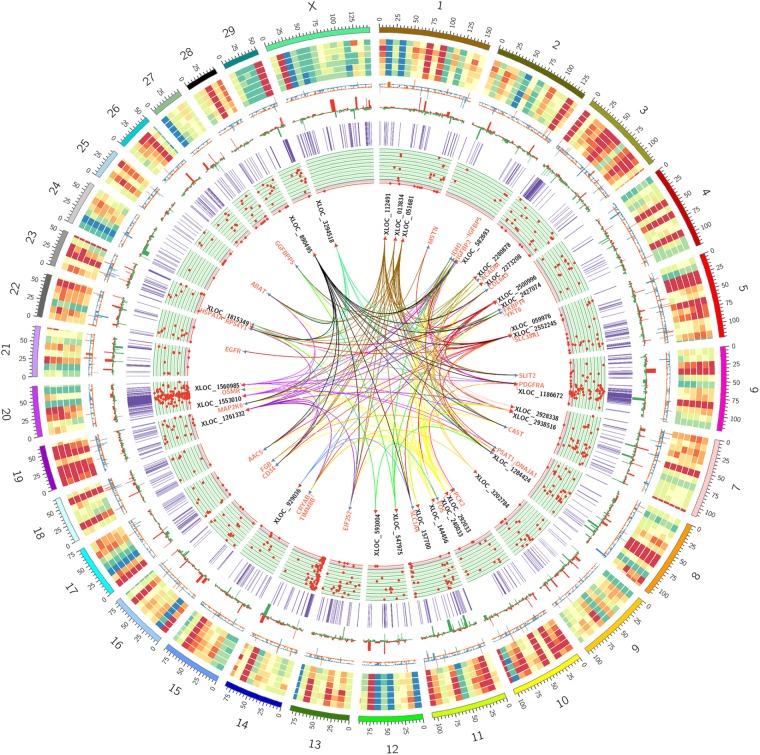
Circos plot of the RNA-seq data, QTL databases and GWAS data. From outside to inside: chromosome number, reads coverage of six samples (three LP samples and three HP samples), mRNAs relative change between HP and LP (orange represents upregulation, blue represents downregulation), lncRNAs relative change between HP and LP (red represents upregulation, green represents downregulation), the location of milk protein QTLs from animal QTL database, the location of significant SNPs on milk protein traits from several previous research, the names of promising lncRNAs and mRNAs pairs associate with milk protein and the *cis/trans* target correlation between promising lncRNAs and mRNAs associate with milk protein.

**FIGURE 6:**
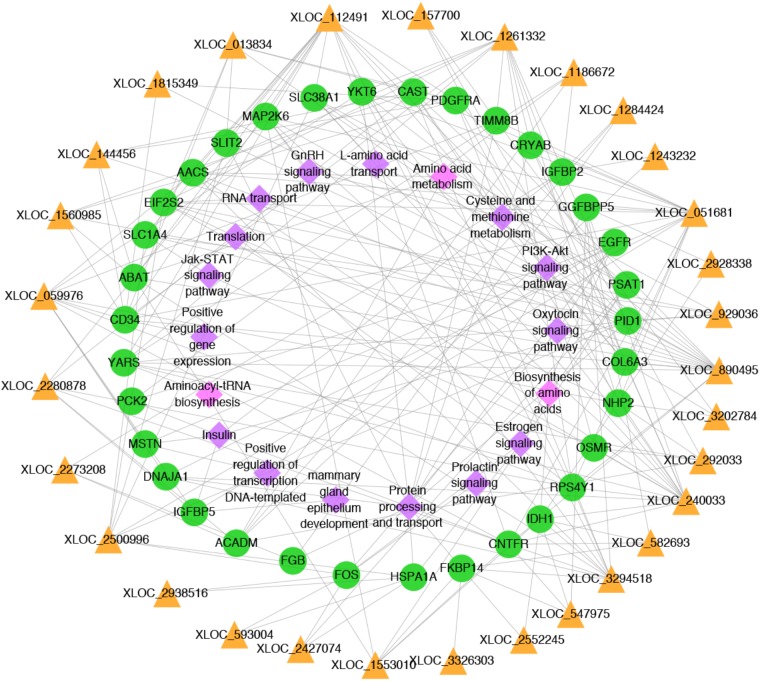
Network plot of candidate lncRNAs, mRNAs and pathways. The yellow triangles, green circles and pink diamonds represent lncRNAs, mRNAs and pathways, respectively.

To better understand the relationship between lncRNAs and milk protein traits, we selectively analyzed the 2,868 lncRNA-mRNA pairs in which both lncRNAs and their neighboring or expression correlated genes were differentially expressed between HP and LP groups. At the same time, the selected target genes should be associated with milk protein metabolism based on reported gene function or their GO and pathway results, and the differentially expressed lncRNAs and their target DEGs should be closed to milk protein QTLs or 972 milk protein SNPs from GWAS studies (**Supplementary Table S3**). The integrated plot is presented in **Figure 5**. It was observed that the majority of QTLs and significant SNPs associated with milk proteins are located in chromosomes 6 and 20. Several differentially expressed lncRNAs and mRNAs were also detected in these regions. According to the integrated study, 30 lncRNAs potentially regulated 34 genes that were involved in milk protein synthesis (**Supplementary Table S14**). For example, *IGFBP2*, a significantly dysregulated milk protein gene (Li C. et al., 2016), the expression of *IGFBP2* was significantly correlated with XLOC_1186672, XLOC_1284424, XLOC_1243232, and XLOC_2273208 simultaneously. EIF2S2, significantly correlated with XLOC_929036, initiated mRNA translation and directly induced protein synthesis. Additional genes are listed in **Table 3**. In addition, we believed that we were able to account for all known target genes involved in milk protein synthesis through various pathways (**Figure 6**).

**Table 3:** The differentially expressed lncRNAs with their potential target genes related to milk protein synthesis.

lncRNA gene	lncRNA transcript	Regulation	Correlated milk protein DEGs
XLOC_2928338	TCONS_4440543	Down	GGFBPP5, IDH1
XLOC_1284424	TCONS_2040856	UP	IGFBP2, FGB, ACADM
XLOC_013834	TCONS_0089292	UP	CAST, ABAT, SLIT2, PDGFRA, CD34, PID1
XLOC_240033	TCONS_0542901	Down	YKT6, CRYAB, SLC38A1, CAST, MAP2K6, GGFBPP5, AACS, EIF2S2, NHP2, CNTFR, DNAJA1, YARS, IDH1, RPS4Y1, COL6A3, OSMR
XLOC_1261332	TCONS_2499784	Down	YKT6, CRYAB, TIMM8B, AACS, EIF2S2, NHP2, CD34, YARS, RPS4Y1, COL6A3, PID1
XLOC_1815349	TCONS_3601672	UP	PDGFRA, CD34, PID1
XLOC_1186672	TCONS_1844622	UP	IGFBP2, PCK2, YARS, ACADM
XLOC_3294518	TCONS_5106036	Down	YKT6, CAST, MAP2K6, ABAT, GGFBPP5, AACS, FKBP14, CNTFR, DNAJA1, IDH1, RPS4Y1
XLOC_059976	TCONS_0458010	Down	CNTFR, YKT6, TIMM8B, AACS, NHP2, IGFBP5, CD34, MSTN, DNAJA1, RPS4Y1
XLOC_547975	TCONS_1098704	Down	GGFBPP5, FKBP14, CNTFR, IDH1
XLOC_2280878	TCONS_4547736	Down	CAST, MAP2K6, IGFBP5, CNTFR, ACADM, RPS4Y1
XLOC_1553010	TCONS_3078081	Down	MAP2K6, ABAT, FKBP14, PSAT1, CNTFR, IDH1
XLOC_582693	TCONS_0918942	Down	GGFBPP5, FKBP14, CNTFR, IDH1
XLOC_144456	TCONS_0207836	UP	CAST, ABAT, PDGFRA, CD34, PID1
XLOC_2500996	TCONS_4893811	UP	SLC38A1, FOS, SLIT2, EIF2S2, SLC1A4, EGFR, HSPA1A, PID1
XLOC_2938516	TCONS_4397837	UP	FOS, HSPA1A
XLOC_2552245	TCONS_3954401	Down	FKBP14, IDH1
XLOC_3202784	TCONS_6404733	Down	TIMM8B, NHP2, COL6A3
XLOC_1243232	TCONS_1975869, TCONS_1991976	UP	IGFBP2, ACADM
XLOC_051681	TCONS_0229895	Down	YKT6, CRYAB, CAST, MAP2K6, TIMM8B, GGFBPP5, AACS, EIF2S2, NHP2, IGFBP5, CD34, MSTN, CNTFR, DNAJA1, RPS4Y1, COL6A3
XLOC_2273208	TCONS_3400346	UP	IGFBP2, FGB, ACADM
XLOC_1560985	TCONS_2392159	UP	CAST, ABAT, PDGFRA, CD34, PID1
XLOC_929036	TCONS_1690898	Down	SLC38A1, EIF2S2, SLC1A4, YARS, PID1
XLOC_292033	TCONS_0432200	Down	CRYAB, NHP2, YARS, ACADM, OSMR
XLOC_2427074	TCONS_4920751	UP	FOS, PSAT1, HSPA1A
XLOC_157700	TCONS_0265438	Down	TIMM8B, NHP2, COL6A3
XLOC_593004	TCONS_1139318	UP	FOS, HSPA1A
XLOC_3326303	TCONS_6686797	Down	FKBP14
XLOC_890495	TCONS_1424422	UP	YKT6, SLC38A1, CAST, MAP2K6, ABAT, SLIT2, AACS, EIF2S2, PDGFRA, SLC1A4, CD34, CNTFR, YARS, RPS4Y1, COL6A3, PID1
XLOC_112491	TCONS_0385499	UP	YKT6, SLC38A1, CAST, MAP2K6, ABAT, SLIT2, AACS, EIF2S2, PDGFRA, SLC1A4, CD34, CNTFR, YARS, RPS4Y1, COL6A3, PID1

*DEGs: differentially expressed genes.*

Notably, lncRNA (XLOC_059976) had a strong positive correlation (*r*^2^= 0.947) with the expression of *CNTFR*, which was identified as a candidate gene for milk protein traits in our previous study (Li C. et al., 2016). To confirm whether XLOC_059976 has coding potential, a search for a Kozak consensus sequence was conducted. However, the sequence (gcc)gccRccAUGG was not observed in XLOC_059976, indicating that there was no efficient and reliable Kozak sequence for initiation of translation (Kozak, 1986). Interestingly, a specific interaction between XLOC_059976 and the upstream region of *CNTFR* was found using the ‘LncTar’ algorithm, which indicated XLOC_059976 could bind to an upstream site of *CNTFR* (**Supplementary Figure S6**). Validation of the result by qRT-PCR revealed that both XLOC_059976 and *CNTFR* were expressed at high levels in the HP group (**Supplementary Figure S7**). Therefore, it is possible that XLOC_059976 may act as a regulatory module by enhancing the expression of *CNTFR* and affecting the secretion of milk proteins.

## Discussion

The mammary gland is the most important organ for the synthesis and secretion of milk and milk proteins. Milk protein synthesis is influenced by thousands of molecules, including lncRNAs. Until now, little is known about the bovine lncRNA transcriptome in mammary gland. In this study, we conducted a preliminary investigation of lncRNA expression profiles in mammary glands of Chinese Holstein cows to assess the potential regulators of milk protein during the lactation period. Consequently, the present work provides an important resource of lncRNAs in mammary gland for future studies.

### The Characteristics of lncRNAs in Mammary Gland

A total of 6,450 multiple-exon lncRNA transcripts were identified corresponding to 5,256 lncRNA genes, including 2,359 known and 4,091 novel lncRNAs transcripts from six mammary samples of Chinese Holstein cows. Most of the detected lncRNAs were expressed at low levels, which implies that lncRNAs and mRNAs have many differences in their biogenesis, processing, stability and spatial-temporal expression patterns (Zhou et al., 2014). The sizes of lncRNAs are smaller than mRNAs probably due to incomplete assembly. RNAs lack an ORF of 360 nt or longer have been classified as putative non-coding RNAs (Dinger et al., 2008). However, 4% of the ORF lengths of mRNAs are observed to be shorter than 120 AAs, which suggest that these short mRNAs may be much more prevalent than previously thought. The low GC content of lncRNAs indicate that they might contain fewer stable base-paired structures, making their primary sequence more accessible for interacting with cellular factors (Niazi and Valadkhan, 2012). PhastCons scores and NONCODE database searches demonstrate that lncRNAs have a less sequence conservation compared to protein-coding genes, although they are much more conserved than random sequences. This moderate conservation for lncRNAs will provide the central argument for lncRNA functionality (Guttman et al., 2009). In general, compared to protein-coding genes, the lncRNAs identified in the present study exhibit significantly lower expression levels, fewer exons, shorter transcripts and ORF lengths, and lower sequence conservation than mRNAs, which is consistent with studies in other species (Cabili et al., 2011; Pauli et al., 2012; Young et al., 2012).

### The Relations Between lncRNAs and miRNAs

lncRNAs may exert their regulatory functions by producing or interacting with small RNAs. For example, lncRNA-H19 hosts miR-675 in its first exon (Cai and Cullen, 2007; Keniry et al., 2012). Our study revealed that 13 lncRNAs contained 8 miRNA precursors, indicating that some lncRNAs could be processed into miRNAs, at least in bovine. Although only 8 miRNAs precursors were identified by our examination, lncRNAs might be an important resource for the identification of novel miRNAs. Whether lncRNAs exert functions by themselves or as precursors of miRNAs is required to be further explored. The target prediction supported a functional network between lncRNAs and miRNAs, and identifying well-established miRNAs that bind to lncRNAs may help infer the functions of lncRNAs. It has been reported that some miRNA may act as important regulators in the process of milk protein synthesis, such as miR-15a (Li H.M. et al., 2012), miR-486 (Li et al., 2015), miR-135 (Ji et al., 2015), miR-101a (Tanaka et al., 2009), miR-152 (Wang et al., 2014), and miR-139 (Cui et al., 2017). Here, 206 lncRNAs were identified having target sites with above miRNAs, implying these lncRNAs may involve in milk protein synthesis. miR-152 can reduce global DNA methylation and the activity of DNMT to reactivate the lactation signal transduction genes *AKT* and *PPARγ* (Wang et al., 2014). miR-139 can suppress β-casein synthesis and proliferation in bovine mammary epithelial cells by targeting the GHR and IGF1R signaling pathways (Cui et al., 2017). XLOC_059976 was predicted to be targeted by both miR-152 and miR-139, implying it might be involved in milk protein metabolism, although further specific validations are required to be confirmed.

### Prediction of lncRNAs Function

Recent studies have indicated that some lncRNAs may act in *trans* and *cis* to regulate the expression of genes (Orom et al., 2010; Cabili et al., 2011; Luo et al., 2013). In this study, we found 31 differentially expressed lncRNAs significantly correlated with 11,161 mRNAs, indicating these lncRNAs may participate in various biological processes in mammary gland. A strong correlation was detected between lncRNAs and their neighboring genes. Taken together, these findings suggest that several lncRNAs may act in *cis* to regulate their neighboring gene expression. After merging the *trans-acting correlation* and neighbor *cis* results, we found 867 lncRNAs expression were significantly correlated with their 979 neighboring genes. Notably, GO functional annotation of *trans-acting correlation* and *cis* results show that the potential target genes of lncRNAs are enriched in transcription regulation, mammary gland development, tyrosine phosphorylation of Stat5 protein and peptide catabolic process. All these terms are related to milk protein synthesis. The important enriched pathways including EIF2 signaling, JAK/Stat signaling, GnRH signaling, PRL signaling, mTOR signaling and growth hormone signaling, are involved in milk protein synthesis. Overall, lncRNAs may affect the synthesis of milk proteins through regulating protein-coding genes in *trans* or *cis*.

Therefore, based on pairs from *cis/trans* target prediction of the differentially expressed lncRNAs combined with gene functions, pathways, milk protein QTL regions plus GWAS results, we are able to identify promising pairs and candidate lncRNAs relevant to milk protein synthesis, transport and metabolism. 30 lncRNAs were predicted to regulate 34 genes affecting milk protein synthesis. PRL is known to activate the phospholipase C-protein kinase C pathway, as well as the phosphorylation of STAT5 proteins, which can enhance the rate of transcription of several milk protein genes (Waters and Rillema, 1989; Wakao et al., 1992). Three DEGs (*FOS, IRF2* and *SOCS2*) were found be involved in PRL signaling pathway (Li C. et al., 2016). It has been reported that the PRL stimulation of protein kinase C may be causally related to the PRL stimulation of *FOS* mRNA accumulation, and protein kinase C activation is essential for all of PRL’s actions on milk product synthesis and mitogenesis (Rillema and Rowady, 1996). The expression of XLOC_2427074, XLOC_2500996, XLOC_2938516 and XLOC_593004 significantly correlated with *FOS* gene imply their important role in milk protein synthesis. It is well-known that insulin and insulin-like growth factor can affect milk protein expression through the activation of STAT5 or regulating amount of translation via mTOR pathway (Burgos and Cant, 2010; Menzies et al., 2010). The CT/GT haplotype of *IGF2* has been proved to be significantly associated with milk protein trait (Bagnicka et al., 2010). The expression of *IGF2* is regulated by six IGF binding proteins (IGFBP-1 to -6) (Pfaffl et al., 2002). Interestingly, we found *IGF2, IGFBP2*, and *IGFBP5* were differentially expressed between HP and LP group (Li C. et al., 2016). IGFBP2 was predicted to correlate with XLOC_1186672, XLOC_1243232, XLOC_1284424 and XLOC_2273208, while *IGFBP5* was predicted to correlate with XLOC_051681, XLOC_059976 and XLOC_2280878. Based on these, we guess that the lncRNA- IGFBP2/IGFBP5 pairs may be involved in insulin-like growth factor pathway and affect milk protein synthesis.

As a novel milk protein associated gene, *CNTFR* is capable of activating multiple downstream signaling pathways, such as AMPK, Jak2-Stat5, MAPK and PI3K-AKT (Weis et al., 1999; Li C. et al., 2016), and their expression appears to be strongly correlated with XLOC_059976. We previously revealed that XLOC_059976 may affect milk protein synthesis by interacting with miR-152 and miR-139. Another possible function of this lncRNA is to regulate the transcription of protein-coding transcripts through binding regulatory region (Rinn and Chang, 2012). Here, we observed a specific binding site for XLOC_059976 located in the 5′ upstream region of *CNTFR*. Based on this specific interaction site, we speculate that XLOC_059976 may enhance *CNTFR* functions through recruiting transcription factors or directly initiating the transcription process, which can affect the downstream process of milk protein secretion. *CNTFR* is the ligand-specific component of a tripartite receptor for ciliary neurotrophic factor (CNTF), which can activate multiple downstream signaling pathways, such as AMPK, Jak2-Stat5, MAPK and PI3K-AKT. All these pathways are critical to milk protein synthesis (Bionaz and Loor, 2011; Tanos et al., 2012). Therefore, XLOC_059976 may play a significant role in the synthesis of milk proteins, and could be a key candidate marker for the selection of milk protein traits. Notably, multiple genes associated with milk secretion could be controlled by single lncRNA. In particular, XLOC_051681 was observed to be correlated with the expression of 16 genes. By contrast, one gene associated with milk protein traits could be regulated by several lncRNAs. For example, *EIF2S2* is believed to be simultaneously targeted by XLOC_051681, XLOC_112491, XLOC_1261332, XLOC_240033, XLOC_2500996, XLOC_890495, and XLOC_929036 (Li C. et al., 2016). The functional roles of lncRNAs are highly complex and diverse. Therefore, our ongoing effort will focus on the function of their target genes, with the expectation that more fundamental information contributing to our understanding of milk protein synthesis and secretion on a molecular level will be obtained.

## Conclusion

Here, we performed a comprehensive analysis of mammary lncRNAs in Chinese Holstein cattle. Bioinformatics approaches were applied to predict the target genes and the potential functions of the differentially expressed lncRNAs. We also sought to explore their roles in milk protein synthesis. The structural characteristics of the identified lncRNAs in bovine are similar to those in other mammalian species. An integrated interpretation of differential lncRNA and mRNA expression reveals that 30 lncRNAs potentially regulate 34 genes affecting milk protein synthesis. As the role of lncRNAs in the bovine mammary gland have not been fully elucidated, our study provides a valuable starting point for future analyses. Moreover, the putative lncRNA XLOC_059976 could be a key candidate biomarker for the prediction of milk protein composition phenotypes, and deserve to be further explored.

## Data Availability

The sequencing data have been submitted to the NCBI SRA, and are accessible through the accession number PRJNA416150.

## Author Contributions

SZ conceived and designed the study, and revised the manuscript. WC performed the RNA related experiments, data analysis, and drafted the manuscript. CL participated in the experimental design, mammary gland biopsy and samples collection. SL conducted the qPCR validation. CZ and HY participated in mammary gland biopsy and samples collection. JS and QZ participated in the result interpretation and paper revision. All authors read and approved the final manuscript.

## Conflict of Interest Statement

The authors declare that the research was conducted in the absence of any commercial or financial relationships that could be construed as a potential conflict of interest.

## Footnotes

1 https://github.com/TransDecoder

2 http://www.mirbase.org/

3 https://www.animalgenome.org/cgi-bin/QTLdb

## Supplementary Material

FIGURE S1 The distribution of reads aligning to genome. **(a–c)** The distribution of reads for HP group in genome. **(d–f)** The distribution of reads for LP group in genome.

FIGURE S2 The number of different class codes for Cufflinks, Scripture, StringTie and Transcomb. The class codes represented the transcripts falls different regions based on the reference annotation you provided. The class code definition (http://cole-trapnell-lab.github.io/cufflinks/cuffcompare/).

FIGURE S3 The predicted secondary structures of lncRNAs transcripts and miRNAs sequences. The secondary structure was created by Vienna RNA package RNAfold web (http://rna.tbi.univie.ac.at/).

FIGURE S4 Seven lncRNAs located in the casein genes cluster on bovine genome. Blue and red box represented lncRNA transcripts and the casein genes, respectively. TCONS_0484809 located in the first intron of *CSN3*.

FIGURE S5 The expression boxplot for neighbor genes of lncRNAs. The red and blue boxplots represent the gene expression within 100 and 10 k upstream/downstream of lncRNAs, respectively. The green boxplot is the expression of all protein-coding genes.

FIGURE S6 Validation of XLOC_059976 and target gene (*CNTFR*) by qRT-PCR. *GAPDH* gene was used as housekeeping internal control. Transcript expression was quantified relative to the expression level of housekeeping using the comparative cycle threshold (ΔCT) method. The data were presented as the mean ± SE (*n* = 3).

FIGURE S7 The combine sites between XLOC_059976 and CNTFR.

TABLE S1 The phenotype information of six Chinese Holstein cattle.

TABLE S2 The concentration and quality information of RNA samples.

TABLE S3 The 12 previous GWAS studies for milk protein traits.

TABLE S4 Summary of sequence reads alignment.

TABLE S5 The annotation of lncRNAs with gtf format.

TABLE S6 The annotation of novel genes with gtf format.

TABLE S7 lncRNA sequences blast against NONCODE database.

TABLE S8 The results of differentially expressed lncRNAs, novel genes and protein-coding genes. **(a)** The differentially expressed lncRNAs results. **(b)** The differentially expressed novel gene results.

TABLE S9 lncRNAs harboring complete precursors of miRNAs.

TABLE S10 The target prediction between lncRNAs and miRNAs. **(a)** The target prediction between all lncRNAs and all miRNAs. **(b)** The target prediction between lncRNAs and milk protein miRNAs.

TABLE S11 Pearson correlations between protein-coding genes and differentially expressed lncRNAs.

TABLE S12 The protein-coding genes within 10 and 100 k upstream and downstream of an lncRNA. **(a)** The protein-coding genes within 10 and 100 k upstream and downstream of lncRNAs. **(b)** The prediction of lncRNA- mRNA pairs by both *cis* and *trans*. **(c)** The expression patterns for differentially expressed lncRNA and their differentially expressed neighboring genes.

TABLE S13 Functional enrichment analysis of the protein-coding genes of lncRNAs in cis and trans. **(a)** The Gene Ontology results of *cis* and *trans* target genes using DAVID. **(b)** The pathway results of cis and trans target genes using IPA.

TABLE S14 The integrate study for differential lncRNA-mRNA pairs.

## Abbreviations

ANXA2: annexin A2
ATF4: activating transcription factor 4
BLAST: basic local alignment search tool
CNS2: casein beta
CNTFR: ciliary neurotrophic factor receptor
CSN1S1: casein alpha s1
CSN1S2: casein alpha s2
CSN3: casein kappa
CYP1A1: cytochrome P450 family 1 subfamily A member 1
DEGs: differentially expressed genes
DHI: dairy herd improvement
EIF4G1: eukaryotic translation initiation factor 4 gamma 1
ENPP5: ectonucleotide pyrophosphatase/phosphodiesterase 5
ERBB2: receptor tyrosine-protein kinase erbB-2 precursor
FPKM: fragments per kilobase of transcript per million mapped reads
FOS: fos proto-oncogene, AP-1 transcription factor subunit
GALE: UDP-galactose-4-epimerase
GAPDH: glyceraldehyde-3-phosphate dehydrogenase
GO: gene ontology
GWAS: genome wide association study
HP: high milk protein
HSPA8: heat shock protein family A member 8
IDH1: isocitrate dehydrogenase (NADP(+)) 1
IGF2: insulin like growth factor 2
IGFBP2: insulin like growth factor binding protein 2
IGFBP5: insulin like growth factor binding protein
ilncRNAs: intronic lncRNAs
IRF2: interferon regulatory factor 2
KEGG: kyoto encyclopedia of genes and genomes
LALBA: lactalbumin alpha
LARP4B: la ribonucleoprotein domain family member 4B
LGB: progestagen-associated endometrial protein
lincRNAs: intergenic lncRNAs
LncNATs: natural antisense lncRNAs
LP: low milk protein
MARVELD1: MARVEL domain containing 1
NGS: next generation sequencing
ORF: open reading frame
PRL: prolactin
qRT-RCR: real-time quantitative reverse transcript polymerase chain reaction
QTL: quantitative trait locus
RIN: RNA integrity number
SNP: single nucleotide polymorphism
SOCS2: suppressor of cytokine signaling 2
ssRNA-seq: strand-specific RNA sequencing
TRIB3: tribbles pseudokinase 3
