# Supplementary material for: Genome Wide Identification of Novel Long Non-coding RNAs and Their Potential Associations With Milk Proteins in Chinese Holstein Cows
Source: Front Genet. 2018 Jul 30;9:281. doi: 10.3389/fgene.2018.00281 (PMC6077245; doi:10.3389/fgene.2018.00281)
Supplement: FIGURE S1 — The distribution of reads aligning to genome. (a–c) The distribution of reads for HP group in genome. (d–f) The distribution of reads for LP group in genome. [file Image_1.PDF]

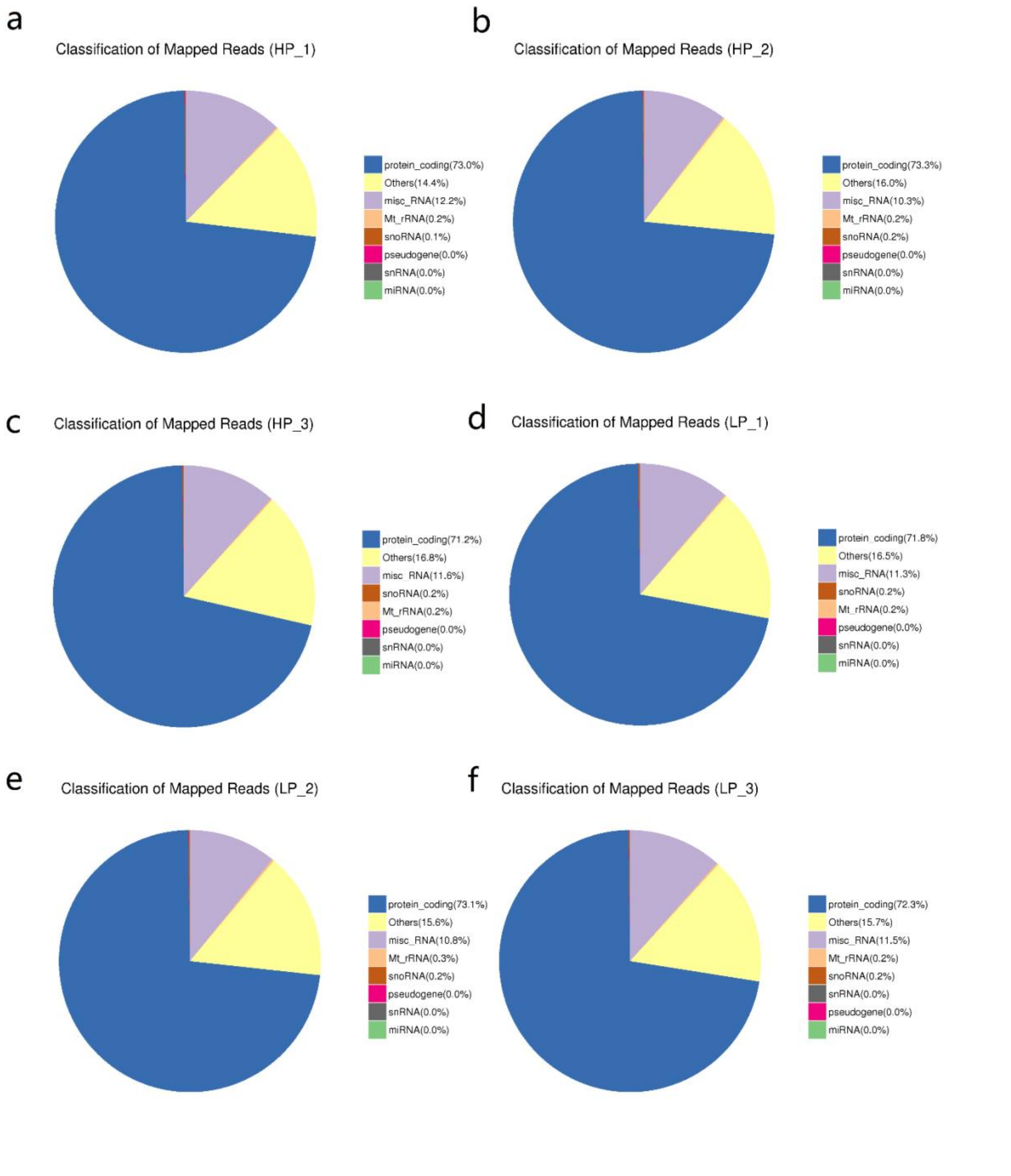

Supplementary Figure 1. The distribution of reads aligning to genome. (a - c) The distribution of reads for HP group in genome. (d - f) The distribution of reads for LP group in genome.
