# Supplementary material for: Genome Wide Identification of Novel Long Non-coding RNAs and Their Potential Associations With Milk Proteins in Chinese Holstein Cows
Source: Front Genet. 2018 Jul 30;9:281. doi: 10.3389/fgene.2018.00281 (PMC6077245; doi:10.3389/fgene.2018.00281)
Supplement: FIGURE S2 — The number of different class codes for Cufflinks, Scripture, StringTie and Transcomb. The class codes represented the transcripts falls different regions based on the reference annotation you provided. The class code definition (http://cole-trapnell-lab.github.io/cufflinks/cuffcompare/). [file Image_2.PDF]

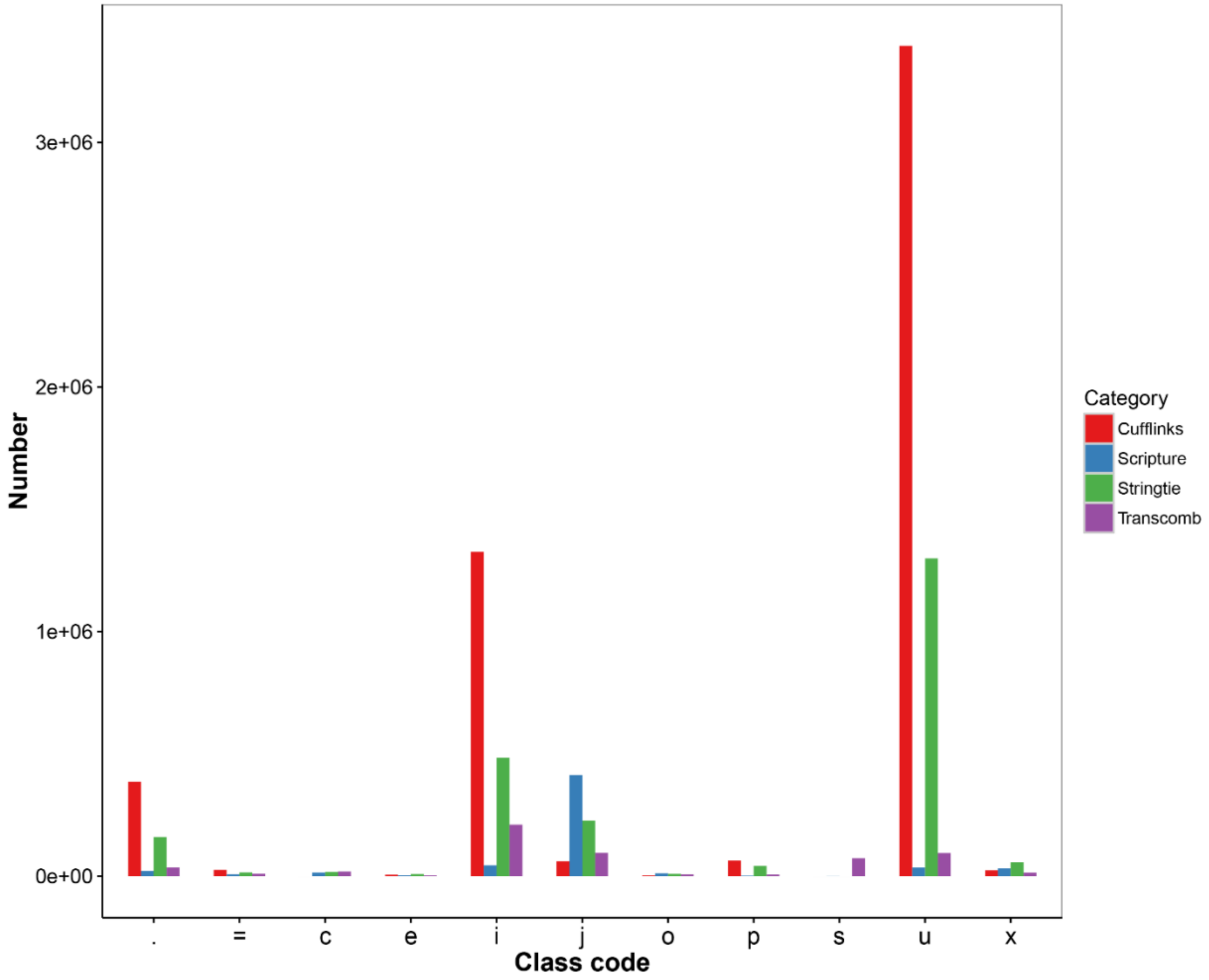

Supplementary Figure S2: The number of different class codes for Cufflinks, Scripture, StringTie and Transcomb. The class codes represented the transcripts falls different regions based on the reference annotation you provided. The class code definition (http://cole-trapnell-lab.github.io/cufflinks/cuffcompare/)
