# Supplementary figures and images for: Genome Wide Identification of Novel Long Non-coding RNAs and Their Potential Associations With Milk Proteins in Chinese Holstein Cows
Source: Front Genet. 2018 Jul 30;9:281. doi: 10.3389/fgene.2018.00281 (PMC6077245; doi:10.3389/fgene.2018.00281)

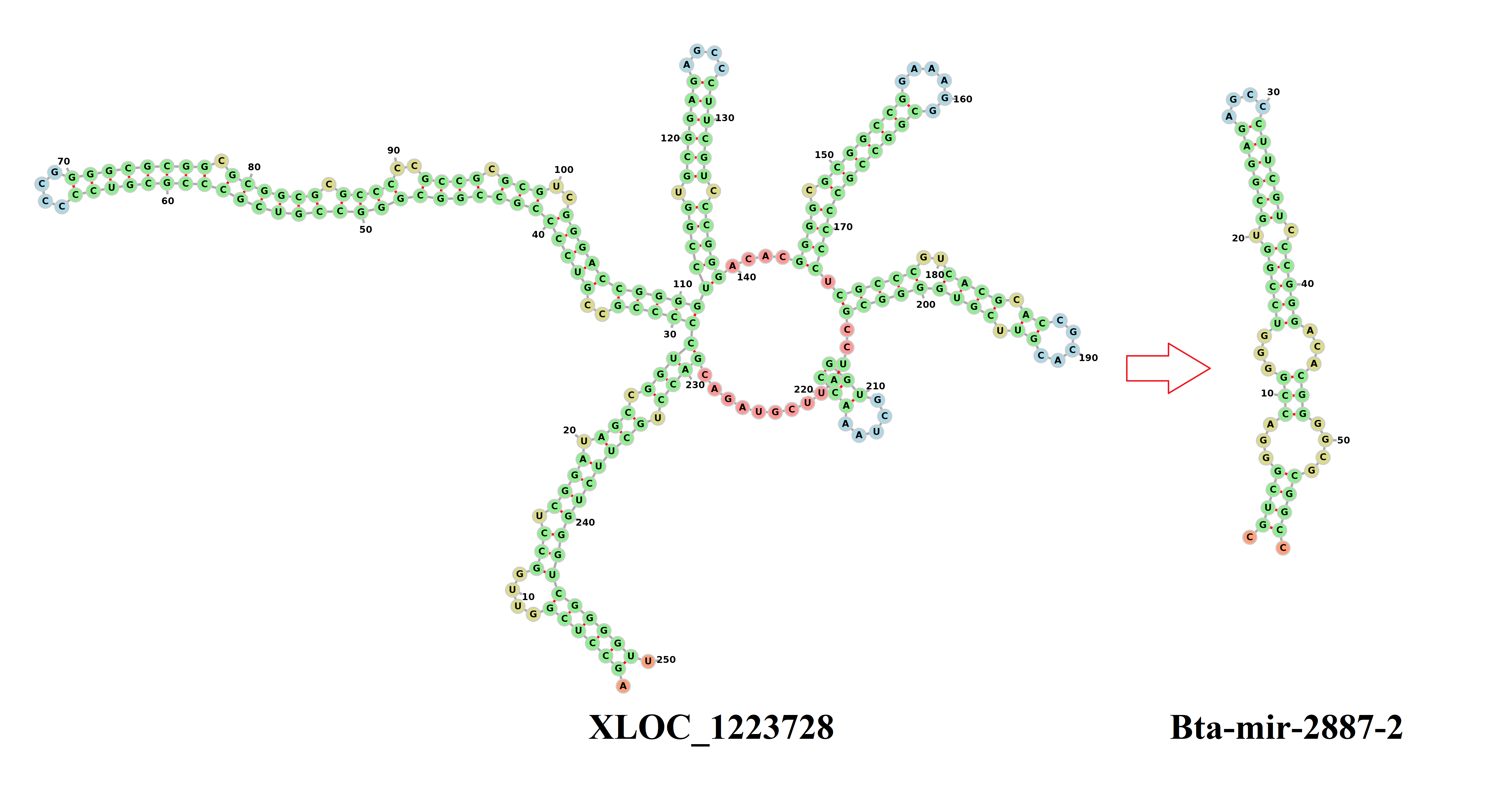

Supplement: FIGURE S3 — The predicted secondary structures of lncRNAs transcripts and miRNAs sequences. The secondary structure was created by Vienna RNA package RNAfold web (http://rna.tbi.univie.ac.at/). [file Image_3.TIF]

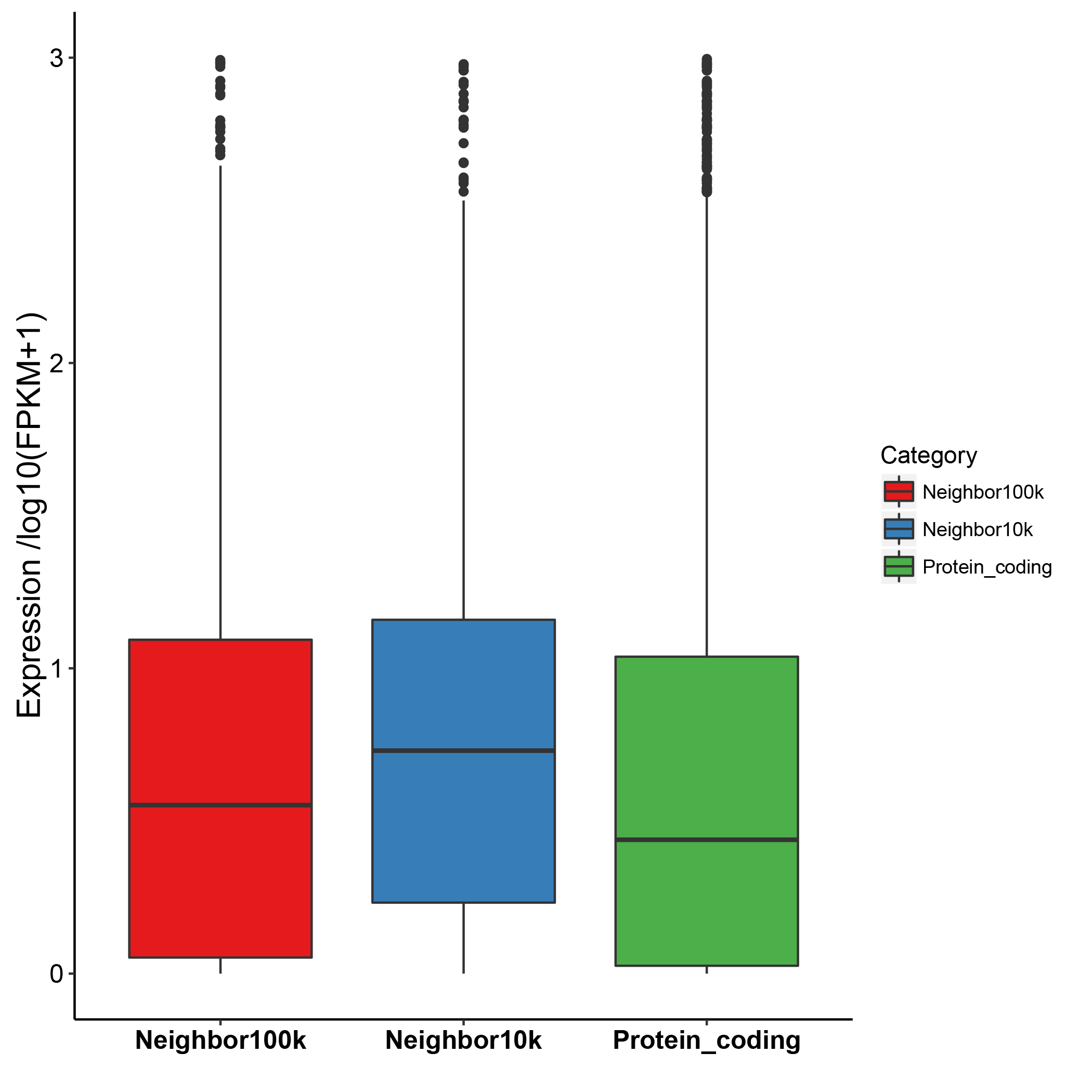

Supplement: FIGURE S5 — The expression boxplot for neighbor genes of lncRNAs. The red and blue boxplots represent the gene expression within 100 and 10 k upstream/downstream of lncRNAs, respectively. The green boxplot is the expression of all protein-coding genes. [file Image_5.TIF]

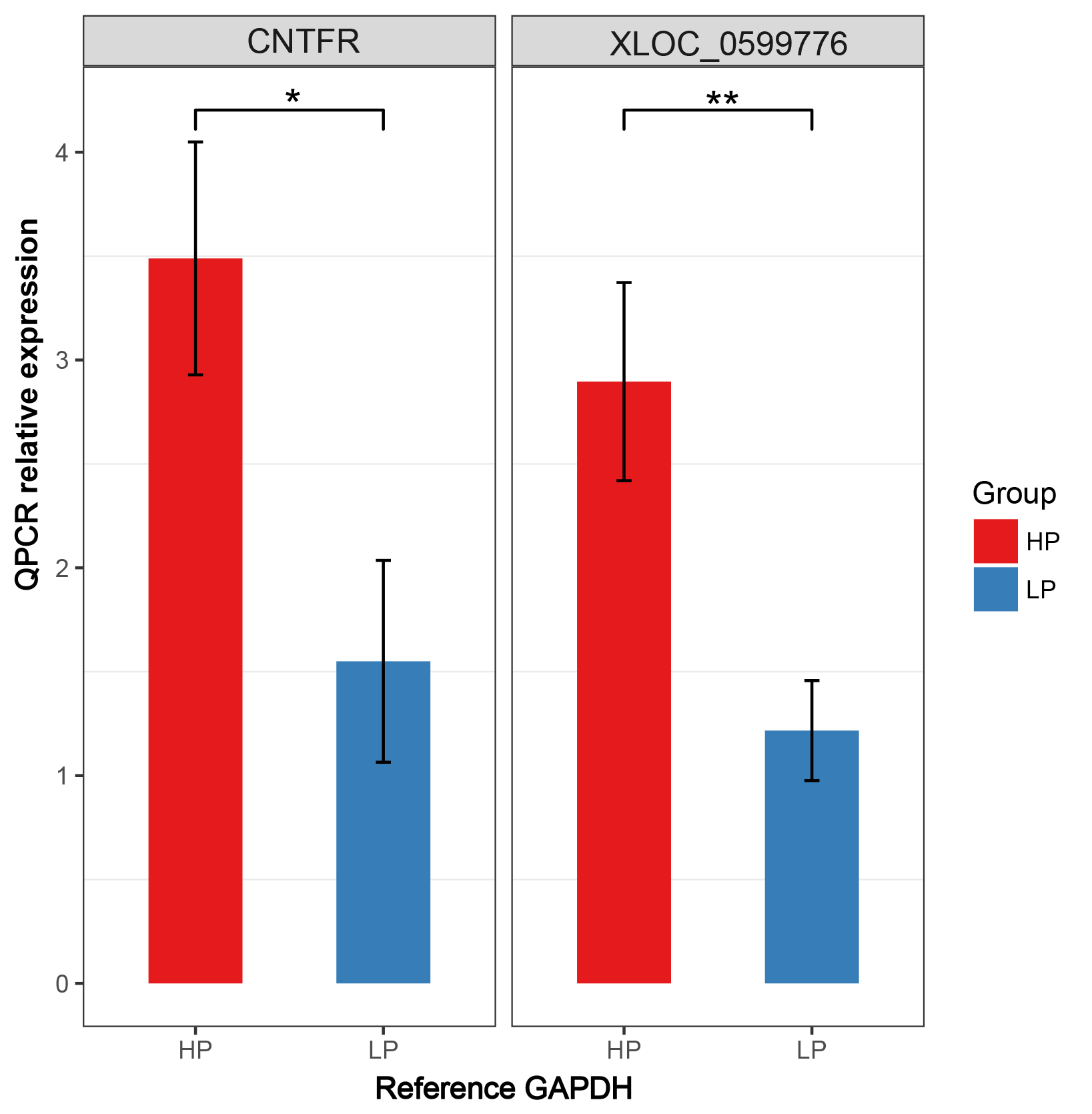

Supplement: FIGURE S7 — The combine sites between XLOC_059976 and CNTFR. [file Image_7.TIF]
