# Supplementary material for: Genome Wide Identification of Novel Long Non-coding RNAs and Their Potential Associations With Milk Proteins in Chinese Holstein Cows
Source: Front Genet. 2018 Jul 30;9:281. doi: 10.3389/fgene.2018.00281 (PMC6077245; doi:10.3389/fgene.2018.00281)
Supplement: FIGURE S6 — Validation of XLOC_059976 and target gene (CNTFR) by qRT-PCR. GAPDH gene was used as housekeeping internal control. Transcript expression was quantified relative to the expression level of housekeeping using the comparative cycle threshold (ΔCT) method. The data were presented as the mean ± SE (n = 3). [file Image_6.PDF]

Query	Length_Query
XLOC_059976	3664

Target	Length_Target
CNTFR	1387

dG	ndG
-78.39	-0.0566

Start_Position_Query	End_Position_Query
1294	2680

Start_Position_Target	End_Position_Target
1	1387

5'
AGTAGGAGGAATAAAAACAAAAGAAATGATAGAAAGAAAGTCAAAGTAGTACTTTACATCAAGAAAATAG
AGTCTTTTATGGGATTTTATGTCTTTAAGAGTGGAAAGAAGAAAGGAATTATTTAGGTGAAGATAAAGGA
GAAGAAAATAAGTGATATCATTGGAAGAATGTGCTAGAGCCTCTCCAAACAGATAAACTGGATTAAAGGT
TCCAGGAACAAATCATGAAACCGGCATAAAACCAAAATCCAGGACTGAGAGGTATGTTGATTTAGTGTAA
TATCATCTTTCATAAGGAAGATGATGGCAGAGAAGAGTTTGCAACTCAAGGTCTAATTTTGACCCCAAAA
GACGAATTTCATCAAGTGGAATGAGAGTGAAGGAAATCTTGAAAGACAGTACTAGGCCATTTTAGTTCTC
CCTGGTAGCCAAGGAGGTAAGGTGAGATGTGTGTCCAGGATTACGGGAAGCTTAACTTCAAATAGTTAAA
TGATACAATATTCATAACCTGAGATTTTATGGGAGACAAGGCTAAAGGAACATGTGATTCTCTTAAGAAT
AAAACTCAGTCAATGCAAATATTTTGGAGTCTAATGAAGAGGAGTTGATGCAAGGATTACAGAGATGCCT
CCAGGGAACTCAGATTTAGAGATGCCCGGCACAGAGGAGGCATAACTAAGAAAGTAGTCCACATATGCAA
ATGCTTTCAAAAGCACAAACCATAAGGCAAAAACTGAGGAATTTGATTACAAAACAAAGACTTATGTTCT
ATAAAGGGCACTATGGACAAAGACAATTCGCAGATGGTAAAATGAAGAAAAAATGTGTTCTGTCTAAAAC
CAACAAAGATGTAATATCATCCTAGAACATATATGGAACTCCTGCAAAGGATCAGGGAAAAGACAGTAAC
CCGATAGGAAAAAAAGTTTCAAATAATATCAACAGGCAATTTAAGAACAGAAAACACTAACATGGATATG
GAAATTTGATCAAAATCATTGGTAATCAAATACAAAGTAAACCAACAATGAGGTATCAGTTCACATCTAT
CAAAAGAGCAAAAATCAGAAATCCATATAATTACTAATGTCAGGAATGTAGAAATGTAGAAACTTGCACT
GCTGCAAGACTGATCGCATCACTTCTGGAAAGCAATCTGGTATAACTCGGATACATTAAATACATTGATA
TGGCCCAGAAATTCCTCTCCTAAGAAATATAAAACAGATTCCTTACAGGTCCAGTAGGGGACTAATAGGA
AAATGTTCTTAGCAGGTTATTTGTAGTGGAGTGGAGCTGTGGGCAACGTGGGTGTTCATCAGTGGAGGCA
TGGATAGATACAATATTGTGAGTGAATACCGCACATCATGGAATAGCAGGCATCAAACTACAGCAACAGA
GTAAATGCTAGCATCTGGTAGCGTGGAGGGAGATGAAAAACGGTGCTCAGTGAAAAAGTAAGGGACAGAA
TGAGGCATAAAACAGTAGCTTTGGAGTACATTTAAGAATAGTGAAGTGAAGTCACTCAGTCATGCCTGAC
TTTTTGTGACCCCATGGATTATGGCCTATCAGGCTCTTCCGTCCATGGGATTTTCCAGGCAAGAATGCTG
GAGTGGATTGCCATTTCCTTCTCCAGGGGATCTTCCCACCCCAGGAATCGAACTCGGGTCTCCCACATTG
CAGGCAGATGCTTTACCGTCTGAGCCACCAGGGAAGCCCAGGGGTAGTTGGCTACATTTAAGAATACATG
CCTACAAAATATTTTCCGAGGGTATATTTAAACACAATTCTAAACACTAGATACGTCTTCCTCAGTGCTT
CAGCTGGTAAAGAACCTGTCTGCCAATGCAGGAGACACATGAGACTCCAGTTTTATCCGTGAGTGGGGAA
GATCCCCTGAAGAAGGGAATGGCTATCATTGGCTTAATCCAATAGAAAGTCAGAGGTAAGGGAATCTGTT
GATGTGGTCCATACAGCTTAGCCTCAAAGCCCAGAGCTGGGTGAAGAGGGTAAGAAATTAAACTGAAGAG
AGAAATAGCTATCTAGGTCGACTGGAAAATAAAAATAAACACGTGTAATCAGATTTGAAATCCCCAAATG
AGATTTTTAAGGGTGTCCTGGGTAAGCCATATATATTAAACTGATTAAGTCACTAATTATGGTGCCTTAG
GATTTTCCTTGAAGGTCCAGGGCTTTGGACACTGACACTCCACTTCTACCAGAACAGCTTTGCTTTTATT
TGGTTAATATAGCAGGAGTCTGCTTAAGATTTCACTGAGGAAACAGAATTATAGCACCACCTATAGTCGG
GCAATGTGCTATGAACTAAAAAATGAATCTCAGAATAGTGGGGAAGACAGAAAAGTGAATTACAGTACAG

TGTCAGGAAAAAAACCATGAAGGGACAGTAAAGAAAGTCCAGTGAACTCCGAAGGGTCAAGGGAACAAGG
AAGGTTTCCTGGAGTCTGTAACTGAAAAGGGAGGTAAGGAAGCTCAGAGAATACTGCCGTTGCCCAAAGG
AGGGAAGAGGAACGCAAGTTACGCAACTCTGAAACGGGCGAAGTCTCCTGGAACGAACGAGGTTACAGAT
CCGCGGACTAGGATGGGCGCTAAAGTGATTTCACGTGATAGGCAAAGACCTACTTAAGCCAGCCTCTAGC
TTTGCTCCGCCCCCATCCCATCTCATCCTTCGTCAAAGGGGCGTGGCATTTAAACAAATGCACTCAGCTG
GGAGGGTATCCCAACCCTGAGGCCCGCGTCACCAGCGCGGGCGGGCGGCTGCGGAGTCTCCCAGAAAAGC
TGGTATCTCCCAGAGTGGTCTGGTTCGGGCTCCCCAGCCCCGGAGGGGCGGCTAAGAAGAAGGCGCTCAC
TGGTTGCTATAGCGGCGATCTAAGAGGCTGGGGAAAGTTGATTGGCCGCAGGTTCGTAGCGCGGACCACG
CCCACCCCTTCCGGCGGCTGCAATCAGGCAAACCTCCATACTATTGTGGGTTCCATTCCAGACCACCAAA
ATAAAAGACTCCTTCCTAAATAGGGACTGAAGACTGTAGGCCTTTCGGGTATAATTCTTGAAATCCTCAA
GTCCTACTGATGTGGTGATACCTGTTACTCAGCCAGTGGGTGTGGTGCTCCGTCGCTCAGTCGTGTCCGA
ATTTGCAGCCTCATGGACTGTAGCCCGTCAGGCTCCTCCATTCATGGAATTTTCCAGGCAAGAATACTGG
AGTGGGGTGCCATTTCCTATTCTAGGGGATCTTCCTGATCCAGGTACAAACCCGTGTCTCTTCATCTCCT
GCACTGGCAGGCAAATTCTTTACTACTTGTTCCACCTGGGAAGCCCTAGTGGGTGCAGCGGAAGGAAATA
ATTGGTGTACTTATGTAGGACCAATAAGGGGAATTATAAAGATGCAGGAGTCAAAGAGGGATGGAAAAGC
ACCCTAGGCTCTCAAGAGTGGGACCAGGTACTACCTTACAGGAACAGGGAAGGGAGCTGTAAAAGAAGGC
CACTTGATAGGGGCTCTGGAACATCAGGGAAGGAGACAGGAAAATAAACACACCCACTCTCTTCCTGCTT
TCCTCTACTCTTACTAACTCTTATTAGTTGAACCCAACCAGAGCTTAGAAGGTAATGAAGGCAGTCAACA
AAAGTCTATCAGGACACAGATCAG 3'

||.....................||.|||.||..||..||........||.........|||........
............||......................................||||.....||.......
............................|||...........||.....|||......|||.........
.......|||..|||...........||......................................||..
................................||.....|||............................
................||....|||||..................||....................|||
.............||..||.||..............||................................
....................................|||..........................||||.
......|||.|||............||...||.........................||...........
...............|||.................||.................................
..............|||....................||....|||........................
.............................||||.|||...........||||.....|||..........
...............||.............|||................||....||.............
..||...................||..............||.......||...................|
||....||..||.............................||.........||......||..||||..
..............|||.......................||||.............||...........
........................||......................||..............||....
...................................||................||...............
.........................|||...........|||............|||.............
||.........................||||.||..||..|||....||....|||.

3'
CTAGTCCTCTGCCGACCGTCACCGTCGTCGCCGTCGGTCCCGGTCTCATTGTCCCTGTGACTGCTTCCTC
CCGCGGCTCCGACGGGGCGGCGACGGGTCGAGGGGCCCCAGTGTCTAGAAGCACCACCCGCCCCCACGGT
CACTCGACCACCACGACCAGCACCAGAGCCCCCGGACCCGGAGTCACCAGTCCACAGCACCGAGGAGGCA
GGTCCCGCACCGCACCCGTCGGTGCGAGGTCAGCGAGGTGCAGGGTTAGAGCAACAGGAACCGGCGGTGG
ACCTACTACATGAGGAACGGTCGCATCCGTAGACACTACCACACACGGCACGGCAGACTGTCGAGGTGTA
CGACGGTGACCAGGTCCTACTCCCCAGCTATCGCGTCTTTCTTGAACTCTCCTTTCCTGAGCCCCAGCCC
GGTCCAGCTGCCCCAGACGGTGCAGTGGAGATCGGCCGCTCCCAACGACCCGTGACCGGCCCGATGGTGT
AAAAGGCCTCCCAGTCCGAAGTGCTACCACTTGAGTAGTTTCCACTACCGACACCGTAACACCGGGTCCC

GCAACGACTGTGAATAGCTCTGGAACATGAACTGCCACCTCTTGTCCACGTACATCGCCTACACCGTCGC
CAAGAACTGCCGGCCCAGGAAGAGTGTCTGGTAGTAAAACCTCGGTACGTCGTGCCAGTGTAACTTCCAC
AACCCCTACATCCACCCTCACCCGTCTACGGTCGACGTCATCTTCGGGAACCCTATTCACAACCTCGCCG
TCGACTCGTGCCCGAGGGCGCCGCCGTTCGGGTGTACCTCTTCGTGGACCACCGCGTCCACGGTCCTCAG
GGCCACCTTCGTCCGCATGTCCGGCGGCACCGGGTCGAGGTCCGAGGCCTCGTGCTCGACTCTCGGCAAC
TCGTCCAGCCCCCGGTCCAGACACGGTAAGTGGGCGGTGCAGTGGCGACGCAGGGTCGACCGTCAGGGTG
TGCCGTCTCAGTGCAGACTCGGGTCCGCGAGCATGACGTGCACCCCTCAGAGGACGCCTGACACAGAGAC
CCGCATCTGGTGCCGTCGTCGTCGTCGCTCGTGCCGTGTCGTCCGGGTGCCCTGTCCTCGTCGGTAGAGA
AGTGGTTGGCGACGTGTGGCTCGTGTAGTTACAGGAGGAGAAGGAGGTCTGGTCCGAGAAGGCCTCTGAA
GACTTTGGTTCCCCCGCCCTACTACACTGTTCCGACCCAAGCGCGGGAGCCTGTCGGCCCCGAGGCGCGG
ATCGAGAAGCTGGCGGGGGCCGTCCCTCTCTCCGTCCCTCGCGCCTCGATCGTGGCGGCGGCCTAGGGCG
GCGGCTCGGGGCGCGACGCGGCCGACCTCGGCGACGGAGGCGACGGCGGCGGCGGGC 5'
